# Effect of Remote Proctoring of the Orthopaedic In-training Examination on Scores

**DOI:** 10.5435/JAAOSGlobal-D-21-00225

**Published:** 2022-02-02

**Authors:** M. Daniel Wongworawat, Maria Incrocci, Christina Fulgaro Crumlish, Joel Klena

**Affiliations:** From the Central Assessments and Examinations Committee, American Academy of Orthopaedic Surgeons (Dr. Wongworawat); the Orthopaedic Surgery Residency Program, Department of Orthopaedic Surgery, Loma Linda University School of Medicine (Dr. Wongworawat); the Examinations and Assessments, American Academy of Orthopaedic Surgeons (Dr. Incrocci and Crumlish); and the Division of Hand and Microvascular Surgery, Orthopaedic Surgery Residency, Geisinger Musculoskeletal Institute (Dr. Klena).

## Abstract

The pandemic has created an opportunity to explore different testing settings. However, transitioning to remote testing raises concerns about cheating. The possibility of cheating raises concern about the integrity of test psychometrics. In 2020, the orthopaedic in-training examination (OITE) was offered using two administration models, with programs having the option to provide physically distanced, in-person, proctored testing or remote testing and online proctoring. We aimed to determine whether scores were higher when the OITE was administered and proctored remotely.

**Methods:**

Residents of allopathic programs from the 2020 administration of the OITE were included. The American Academy of Orthopaedic Surgeons database of examinees and deidentified aggregate scores were compared. The primary outcome variable was the number of items answered correctly. Of 275 questions and after psychometric analysis, eight questions were excluded, leaving 267 questions as the denominator for percent correct. The mean number of items answered correctly was compared between the group with in-person proctoring and the group with virtual, digital proctoring using the two-sample Student *t*-test.

**Results:**

A total of 4405 examinees consisted of two cohorts: 1834 residents (42%) took the OITE with in-person proctoring and 2571 residents (58%) completed the test in the remote testing and proctoring models. No difference in mean scores was observed between in-person proctored and remotely proctored examinees (in-person: 162.98 ± 21.11, remote: 162.22 ± 22.04, mean difference: −0.75 [95% CI, −2.04 to 0.53]; *P* = 0.25).

**Discussion:**

Remote testing with virtual proctoring has become more widely used. There was no difference in scores and no evidence of enough cheating to change the “curve” of the OITE. Knowing that there was no evidence of sufficient cheating to change overall test psychometrics, programs and residents can be reassured that the OITE remains a valid educational instrument even when administered remotely.

The Orthopaedic In-Training Examination (OITE) was the first of its kind, pioneering national knowledge assessment of residents in specialty training. After its authorization by Clinton Compere, then President of the American Academy of Orthopaedic Surgeons (AAOS), the OITE was first administered to 1118 residents in 171 training centers in November 1963, and it has been administered annually since.^1^ The results of each resident's performance allow for comparisons within a program and comparisons between programs. These results have been used to improve curriculum and guide study, with program directors often imposing consequences for poor performance, such as assigned remediation and reprimand.^2^ Initially being a paper-based examination, administration transitioned to electronic versions through mailed software files on compact discs. In 2012, the AAOS transitioned the examination to a web-based format, with testing generally scheduled on the second Saturday of November, with additional testing opportunities allowed on the preceding Friday and following Sunday and Monday to allow scheduling flexibility.

The pandemic that circled the globe in 2020 challenged testing norms. Although the procedures of the OITE have specified in-person proctoring since its inception, physical distancing mandates forced many educators to shift to online testing. Transitioning to remote testing raises concerns about cheating.^3^ The possibility of cheating also raises the level of concern about remote digital proctoring surveillance and the integrity of test psychometrics because they relate to individual scores and percentiles.^4^

In 2020, the OITE was offered using two administration models, with programs having the option to provide physically distanced, in-person, proctored testing or remote testing and online proctoring. We were interested in determining whether test scores were higher when the OITE was administered and proctored remotely. Second, we examined test score differences between in-person and remote testing, as stratified by training year.

## Methods

Institutional Review Board exempt determination was received. We included residents from the 2020 administration of the OITE from allopathic programs. Excluded were examinees from international and nonallopathic programs. The AAOS database of examinees and deidentified aggregate scores were compared.

The primary outcome variable was the number of items answered correctly. This was used as a proxy to measure the level of test difficulty. For the 2020 administration of the OITE, 275 questions were presented, and after psychometric analysis, eight questions were excluded, leaving 267 questions as the denominator for percent correct.

Aggregate data from all examinees from allopathic programs were evaluated for normality by visual inspection of histograms. The mean number of items answered correctly was compared between the group with in-person proctoring and the group with virtual, digital proctoring using the two-sample Student *t*-test with assumptions of unequal variance. Because these data represent all examinees, population statistics were used. The significance level was set at *P* < 0.05.

## Results

A total of 4405 examinees consisted of two cohorts: 1834 (42%) residents took the OITE with in-person proctoring and 2571 (58%) residents completed the test in the remote testing and proctoring models. By training year, there were 860 residents (20%) in the first year, 907 (21%) in the second year, 901 (20%) in the third year, 877 (20%) in the fourth year, and 860 (20%) in the fifth year.

Overall, there was no difference in mean scores between in-person proctored and remotely proctored examinees (in-person: 162.98 ± 21.11, remote: 162.22 ± 22.04, mean difference: −0.75 [95% CI, −2.04 to 0.53]; *P* = 0.25). When stratified by training year, there were no differences between in-person and remote examinees' scores (Table 1).

**Table 1:** Number of Items Answered Correctly of 267 Items

	In-person mean ± SD	Remote mean ± SD	Mean Difference (95% CI)	*P*
Overall	162.98 ± 21.11	162.22 ± 22.04	−0.75 (−2.04 to 0.53)	0.25
PGY-1	136.32 ± 14.80	134.42 ± 15.54	−1.90 (−3.95 to 0.16)	0.07
PGY-2	153.95 ± 15.38	152.45 ± 15.47	−1.50 (−3.53 to 0.53)	0.15
PGY-3	168.81 ± 13.35	167.04 ± 14.64	−1.77 (−3.62 to 0.07)	0.06
PGY-4	174.71 ± 13.58	175.40 ± 12.37	0.690 (−1.07 to 2.44)	0.44
PGY-5	180.97 ± 12.51	181.92 ± 11.92	0.956 (−0.71 to 2.62)	0.26

PGY = post-graduate year, SD = standard deviation, CI = confidence interval

## Discussion

Remote testing with virtual proctoring has become more widely used. The pandemic forced even greater adoption of remote administration of examinations. The 2020 testing circumstances created a unique circumstance where we had two concurrent cohorts: in-person and remote. Therefore, we were able to directly compare test performance between the two groups. We found that there were no differences in scores, and there was no evidence of cheating that was enough to change the “curve” of the OITE.

Several self-reported surveys have explored the prevalence of cheating among medical students. In a 1980 survey of medical students at two US medical schools, 87% of the students admitted to cheating in undergraduate school, with 58% admitting to doing so at least once in medical school.^5^ The definition of cheating included obtaining answers for an examination not yet taken from classmates who had already done so. In a 1996 survey of medical students at Johns Hopkins, 23% of the students admitted to cheating in medical school: Copying answers from classmates and using unauthorized materials on examinations were among the most common occurrences.^6^ A 2010 survey reported similar results for 4,400 students at seven medical schools with 27% responding that they had engaged in dishonest behavior.^7^ A 1996 survey of students at 31 US medical schools found that while only 4.7% of the students admitted to cheating, 39% reported directly witnessing cheating by others.^8^

Several studies outside of medicine have sought to empirically evaluate the integrity of online examinations and have reported varying findings. In a study of economics students, Harmon and Lambrinos^9^ compared proctored and nonproctored online examination results and through statistical analysis concluded that the nonproctored format resulted in cheating. Similar to our findings, two follow-up studies by Harmon and Lambrinos found no such increased incidence of cheating with a nonproctored online format.^10,11^ Evidence exists that when students are invested in the value of what they are learning and they believe that the manner in which they are being assessed is relevant, the prevalence of cheating declines.^12-14^ Regarding orthopaedic residents taking the OITE, recognition of the importance of the examination as a predictor of success on their written boards may be a sufficient disincentive to cheat.

Our findings should be interpreted in light of some limitations. The data represent one year's administration, but it allowed us to compare concurrently, rather than against historic control subjects. Another limitation is that there may be variations in the level of direct supervision with in-person proctoring. Other than each program's attestation, there was no formal mechanism to ensure uniformity of proctoring practices. This study's primary outcome uses numbers answered correctly as a proxy for test difficulty. Although this may not be a perfect measure of difficulty, this measure is the closest metric available for comparisons of cohort performance.

When compared with in-person testing, remote administration of the OITE with virtual proctoring has similar face validity. Knowing that there was no evidence of sufficient cheating to change overall test psychometrics, programs and residents can be reassured that the OITE remains a valid educational instrument. A reminder that the OITE's purpose is an educational tool to identify gaps and help the individual gear toward the board examination, rather than a punitive instrument, may disincentivize residents from cheating.
